# The Economic Consequence of Increased Health Care Resource Utilization in Maryland Associated With a Major Wildfire Smoke Event

**DOI:** 10.1016/j.chest.2026.01.034

**Published:** 2026-03-24

**Authors:** Zafar Zafari, Mary E. Maldarelli, Clayton H. Brown, Madhurika Situt, Colleen Reilly, Rozalina McCoy, Martha Jurczak, Amir Sapkota, Katarina Zeder, Warren D’Souza, Bradley A. Maron

**Affiliations:** a University of Maryland-Institute for Health Computing, Bethesda, MD; b Department of Practice, Sciences, and Health Outcomes Research, University of Maryland School of Pharmacy, Baltimore, MD; c Department of Medicine, University of Maryland School of Medicine, Baltimore, MD; d University of Maryland Medical System, Baltimore, MD; e Department of Geographical Science, University of Maryland, College Park, MD; f Division of Pneumology, Medical University of Graz, Austria

**Keywords:** cardiopulmonary diseases, economic burden, wildfires

## Abstract

**Background:**

Transcontinental wildfire smoke migration originating from remote areas resulted in consecutive days of high concentrations of particulate matter with aerodynamic diameter < 2.5 μm (PM_2.5_) in Maryland in June 2023.

**Research Question:**

What are the economic and cardiopulmonary effects of migrating wildfire smoke exposure during June 2023 in Maryland?

**Study Design and Methods:**

Using electronic health record data from the University of Maryland Medical System, we identified cardiopulmonary clinical encounters. We developed 2 regression models (combining a logistic regression and a generalized linear model with a gamma distribution and log link) and a nonparametric bootstrapping to estimate the incremental cost of cardiopulmonary encounters during 6 days defined by unhealthy PM_2.5_ concentration (hotspot days) in June 2023 in Maryland relative to calendar-matched control days in 2018 and 2019. We also provided 10-year cost projections under different future wildfire scenarios and modeled the preventable burden using respirators.

**Results:**

The final sample included 5,273 cardiopulmonary encounters. The total cost accrued by increased cardiopulmonary encounters during the 6 hotspot day exposure compared with control days was $2,435,513 (95% uncertainty interval [UI], $62,452-$5,266,838). Under the expected scenario (2 wildfires in 10 years), the projected incremental cost of wildfire smoke-related cardiopulmonary encounters was $6,379,406 (95% UI, $122,412-$13,837,150). However, modeling the use of respirators with 50%, 75%, and 95% effectiveness by high-risk individuals was associated with an expected net cost saving of $758,274, $1,162,649, and $1,486,150, respectively.

**Interpretation:**

Our results shows that a major medical system in Maryland experienced substantial health care economic burden over 6 days that was characterized by increased wildfire smoke. Because Maryland uses a fixed global budget system, our findings have implications for preventive strategies that mitigate economic burden incurred in the context of wildfire smoke events.

**Take-Home Points**

**Research Question:** What is the economic consequence of increased cardiopulmonary health care resource utilization associated with migrating wildfire smoke?

**Results:** The 6 smoke-related hotspot days generated an estimated $2.4 million increase in cardiopulmonary encounter costs (95% uncertainty interval, $62,452-$5.27 million).

**Interpretation:** Our results show that our health system, which operates under a fixed, global budget, experienced a significant and unexpected economic burden from migrating wildfire smoke exposure.

In the summer of 2023, Canada experienced a record-high wildfire season for intense and destructive wildfires that consumed > 100,000 hectares of land.^1^ These large-scale wildfires caused a smoke plume that migrated over approximately 2,100 miles to the US East Coast. Although wildfires are not a typical natural hazard in the State of Maryland, infiltrating smoke from wildfires resulted in a statewide exposure to polluted air.^2^ Wildfire smoke contains particulate matter with aerodynamic diameter < 2.5 μm (PM_2.5_), exposure to which is associated with increased cardiovascular events,^3^ including out-of-hospital cardiac arrests^4^ and exacerbation of chronic respiratory conditions, such as asthma and COPD.^5^^,^^6^

In addition to influencing clinical burden of disease,^7^, ^8^, ^9^, ^10^ increased health care utilization associated with migrating wildfire smoke may have significant financial implications for health care systems, particularly those operating under a fixed global budget. For example, the Innovation Center of the Centers for Medicare & Medicaid Services is implementing the Advancing All-Payer Health Equity Approaches and Development (AHEAD) model.^11^ AHEAD is a total cost of care model, in which participating hospitals receive a fixed global budget. The overall goal of AHEAD is to control growth of health care expenditures while improving efficiency and quality of care.^11^ In AHEAD’s model, it is essential for hospitals to prioritize investments in preventive strategies; thus, events such as wildfires may have significant financial implications for these hospitals.

In this study, we aimed to quantify the cost of increased cardiopulmonary utilization associated with migrating wildfire smoke. We used a unique clinical data infrastructure from the University of Maryland Medical System (UMMS). Additionally, we projected the economic burden of future similar wildfire events and modeled how much of the burden can be potentially prevented by use of N95 respirators.

## Study Design and Methods

### Wildfire Smoke and Clinical Outcomes Analyses

Using data from the Hazard Mapping System from the National Oceanic and Atmospheric Administration^12^, ^13^, ^14^ and the Environmental Protection Agency (EPA)’s Air Quality System,^15^ we calculated average daily wildfire-related PM_2.5_ levels (methodology details are provided in e-Appendix 1).

We used the threshold for PM_2.5_ levels representing unhealthy air quality (24-hour average > 35 μg/m^3^) according to EPA National Ambient Air Quality Standards. This threshold corresponds to the Air Quality Index category for Unhealthy for Sensitive Groups (Air Quality Index > 100).^16^ Using this threshold makes the recommendations more policy relevant for operational responses to wildfire smoke and the results directly interpretable for hospital planners.

This approach identified 6 calendar days in June 2023 with PM_2.5_ levels above the EPA-designated unhealthy threshold (hotspot days). The UMMS Electronic Health Record was used to identify all encounters due to cardiopulmonary conditions (encounters included outpatient visits, emergency department visits, and hospitalizations) among patients of all ages during the hotspot days compared with orthologous days in the control years. Orthologous hotspot days were defined as control days in June 2018 and 2019 matched to June 2023 hotspot days by weekday and week of the month, within a fixed 4-week window (first Saturday to last Friday of June). This alignment preserves seasonal timing and weekday patterns while ensuring comparable calendar periods without wildfire smoke exposure. This study was approved by the University of Maryland institutional review board (IRB approval No. HP-00107584).

Unlike the original report,^10^ we adjusted for hospital-specific effects across the 12 hospitals. This enabled within-hospital comparisons over time and improved internal validity because hospitals differ in patient populations, baseline utilization, and capacity—differences that could otherwise confound results across years.

We aimed to include a comprehensive set of cardiopulmonary conditions, encompassing cardiac non-heart failure, heart failure, and respiratory diseases. Our International Classification of Diseases, 10th Revision codes selection aligned with cardiovascular and respiratory outcomes used in previous related studies (codes provided in e-Table 1).^7^, ^8^, ^9^ The baseline characteristics of cardiopulmonary encounters are presented in e-Table 2.

### Building the Economic Impact Model

We used 2 regression models for our estimation. In the first model, we predicted the probability of a cardiopulmonary encounter occurring on hotspot days using a generalized linear model with binomial distribution. This allowed us to calculate the probability of a cardiopulmonary encounter occurring on hotspot days of 2023 against orthologous days in the control years. We combined all cardiopulmonary encounters, encompassing hospitalizations, emergency department visits, and outpatient visits to enhance statistical power. In the second model, we conducted a difference-in-differences (DID) analysis using a generalized linear model with a gamma distribution and log link to compare the cost of each cardiopulmonary encounter between hotspot and nonhotspot days in the wildfire year (2023) vs control years. Analogous to a conventional DID design, the treated units are calendar-position days that experienced wildfire smoke exposure in June 2023, with their orthologous days in June 2018 to 2019 representing pretreatment observations of the same units. Time is divided into a baseline period (June 2018-2019) and a wildfire year (June 2023), with the post indicator defined as an indicator for the wildfire year. The actual wildfire smoke exposure is identified by the interaction between the hotspot indicator and the post indicator. Details on the regression analyses, gamma model selection, and parallel-trends testing for the DID analyses are provided in e-Appendix 2.

For the 2 models, we adjusted for weekday, facility, age (continuous variable), sex (male vs female), race/ethnicity (categories as Black, Asian, and a category for other races into which categories of Native Hawaiian or other Pacific Islander, American Indian or Alaskan Native, two or more races, or other were combined; reference was White), smoking status (categories as patient who formerly smoked, patient who smokes lightly, patient who smokes heavily, and patient who actively smokes with intensity unknown; reference was patient who never smoked), and BMI (as a continuous variable).

We conducted a complete-case analysis by including nonmissing observations in the main analysis (missing values accounted for approximately 9.5% of the data). Additionally, we performed multiple imputations for missing covariate values and reran the analyses to assess the robustness of our findings.

### Estimating Marginal Costs

We estimated marginal direct medical costs using a nonparametric bootstrap with 1,000 replicates, refitting both models in each replicate and computing marginal costs via g-computation. The g-computation involved taking the difference between the expected number of encounters and the expected cost of encounters between the observed hotspot days and the orthologous days. The direct cost included medical cost of a cardiopulmonary encounter. Indirect costs were incorporated via a Monte Carlo simulation; in each bootstrap iteration, we drew annual wages from a gamma distribution (mean, $75,585; based on Bureau of Labor Statistics data^17^), with a 95% uncertainty interval (UI) extending ± 5% of the mean, and combined these draws with bootstrap-sampled lengths of stay (mean, 5 days in our data) to compute productivity and leisure losses. In the base case, we assumed 8 hours of productivity loss and 8 hours of leisure loss per hospitalization day, valued at the hourly wage^18^, ^19^, ^20^, ^21^ (additional details in e-Appendix 3). We prespecified a directional hypothesis—that wildfire smoke exposure increases total costs—based on prior evidence that increases in PM_2.5_ lead to higher spending.^22^ Accordingly, we reported both 1- and 2-sided statistical inference, as suggested by Ruxton and Neuhäuser.^23^

All costs were adjusted to 2024 US dollars. To increase the generalizability of our findings, we conducted 1-way sensitivity analyses for the mean baseline direct medical cost of a cardiopulmonary encounter, the mean annual wage of an American, the length of stay per encounter, and the number of hours lost per cardiopulmonary encounter (e-Appendix 3).

### Projecting Economic Effects of Future Wildfires

For projection of future wildfire events, we first used a Bayesian process using 10-year historical data (2014-2023) from the EPA Air Quality System^15^ and the National Oceanic and Atmospheric Administration’s Hazard Mapping System^12^^,^^24^ to calculate the mean baseline frequency. We accounted for changes in frequency and duration of future events using data from Westerling.^25^ We used a Poisson distribution with the updated frequency mean for future events to define 4 projection scenarios for the next 10-year number of wildfires: scenario A (minimum)—same number of wildfire events as in the past 10 years; scenario B (expected)—mean of the Poisson distribution; scenario C (intermediate)—midpoint between scenarios B and D; and scenario D (worst case)—97.5th percentile of the Poisson distribution. For each simulated future event, duration was scaled relative to the baseline period by applying the 50% per-decade increase, adjusted for the calendar year of occurrence (analytical details are provided in e-Appendix 4).^25^

To project costs of the future wildfires, we also accounted for population growth in Maryland using data from the Maryland Department of Planning.^26^ We assumed that the prevalence of patients with cardiopulmonary disease would change in direct proportion to population changes (e-Appendix 4). We also accounted for real medical cost growth using a 1.91% annual real growth rate derived from published literature.^27^ In the base case analysis, we applied a 3% discount rate to future costs.^18^ In a sensitivity analysis, we also reported the results without any discounting and with a discounting rate of 5%.

### Preventable Cardiopulmonary Costs of Migrating Wildfire Smoke

We modeled the potential net cost savings of providing N95 respirators to high-risk patients with cardiopulmonary disease during hotspot days. High-risk individuals were defined as those with a cardiopulmonary encounter in our medical system in the prior year. Among patients with a cardiopulmonary encounter during the smoke exposure period (June 2023), we calculated the proportion who also had a cardiopulmonary encounter in the previous year. We then assumed that this proportion of the attributable cost of smoke exposure could be prevented if hospitals provided respirators to all high-risk individuals identified from the previous year’s records. We used a bulk respirator cost of $0.78 (2024 US dollars)^28^ and included replacement costs for subsequent events. Based on the literature, we assumed mask effectiveness values of 50%, 75%, and 95%.^29^ More details are provided in e-Appendix 5.

We additionally modeled a sensitivity analysis for the removal of respirators in indoor spaces. Parameterizing a log-normal distribution for the infiltration factor with a mean of 0.23 and 25th and 75th percentiles of 0.16 and 0.36,^30^ we calculated the probability that time spent indoors would be exposed to PM_2.5_ levels exceeding the EPA threshold of 35 μg/m^3^. We then adjusted the respirator protection effect based on average indoor time and the probability of indoor exposure exceeding the EPA’s unhealthy threshold during hotspot days (details are provided in e-Appendix 5).

## Results

The results of 2 regression models are reported in Table 1. The final sample included 5,273 cardiopulmonary encounters during June 2023 and June 2018 to 2019. The adjusted odds of a cardiopulmonary encounter occurring on hotspot days in the wildfire year 2023 relative to the orthologous days in the control years increased statistically significantly by 22% (*P* = .022) (Table 1). The expected cost of a cardiopulmonary encounter occurring on hotspot days relative to control days increased by 11% (*P* = .384). The results from the multiple imputation sensitivity analysis were consistent with the complete-case analysis (e-Table 3). The results of 2 regression models for hospitalizations, emergency department visits, and outpatient visits separately are presented in the e-Tables 4-6.

**Table 1:** Results of the 2 Regression Models Estimating the Economic Effects of Migrating Wildfire Smoke on Cardiopulmonary Health Care Encounters

Variable	Estimate	SE	*P* Value
Model 1 (predicting rate of cardiopulmonary events occurring on hotspot days)			
Intercept	−1.400	0.257	< .001
Year (2023 vs 2018/2019)	0.204	0.089	.022
Demographics			
Age	0.003	0.002	.161
Sex (male vs female)	0.077	0.077	.315
Race/ethnicity (reference: White)			
Black	0.009	0.090	.918
Asian	0.679	0.316	.032
Other^a^	0.419	0.218	.055
Smoking (reference: patient who never smoked)			
Patient who formerly smoked	−0.067	0.096	.489
Patient who smokes lightly	−0.168	0.255	.511
Patient who smokes heavily	−0.069	0.122	.576
Patient who smokes, intensity unknown	0.036	0.129	.778
BMI	0.001	0.005	.897
Model 2 (predicting unit cost of cardiopulmonary events falling on hotspot days)			
Intercept	9.418	0.173	.000
Year (2023 vs 2018/2019)	0.088	0.066	.184
Hotspot (treated unit indicator)	−0.038	0.084	.647
Demographics			
Age	0.001	0.002	.482
Sex (male vs female)	0.112	0.051	.027
Race/ethnicity (reference: White)			
Black	−0.128	0.060	.033
Asian	0.069	0.219	.753
Other^a^	−0.216	0.149	.147
Smoking (reference: patient who never smoked)			
Patient who formerly smoked	0.049	0.063	.438
Patient who smokes lightly	−0.366	0.156	.019
Patient who smokes heavily	−0.130	0.079	.098
Patient who smokes, intensity unknown	−2.657	0.092	.000
BMI	0.001	0.003	.668
Hotspot × year (DID estimator)	0.105	0.121	.384

All the models adjusted for weekday and 12 hospital facilities, including Baltimore Washington Medical Center, Charles Regional Medical Center, Harford Memorial Hospital, University of Maryland Capital Region Health, University of Maryland Shore Medical Center at Chestertown, University of Maryland Shore Medical Center at Dorchester, University of Maryland Shore Medical Center at Easton, University of Maryland Medical Center, University of Maryland Medical Center Midtown Campus, University of Maryland Rehabilitation and Orthopedic Institute, University of Maryland St Joseph Medical Center, and Upper Chesapeake Medical Center. The coefficients represent the conditional effects on the log-odds of experiencing a cardiopulmonary encounter on a hotspot day (model 1, logit model) and on the log of expected encounter costs (model 2, gamma model). For instance, for the sex variable, the log-odds increased by 0.077 and the log expected costs increased by 0.112 for male indivudals compared with female individuals. DID = difference-in-differences.

a Categories of Native Hawaiian or other Pacific Islander, American Indian or Alaskan Native, two or more races, or other were combined into the other category.

### Cost Burden for 12 UMMS Facilities During Hotspot Days

The incremental total cost of hotspot days during June 2023, estimated using a nonparametric bootstrap with g-computation, averaged $2,435,513. Under the 1-sided statistical inference (reflecting the prespecified hypothesis that wildfire smoke increases cost), the 95% lower uncertainty bound was $372,790 for total cost. The mean incremental direct cost was $2,197,133 with a 95% lower uncertainty bound of $240,525, and the mean incremental indirect cost was $238,380 with a 95% lower uncertainty bound of $52,426. Under the 2-sided inference, the mean incremental total cost was $2,435,513 (95% UI, $62,452-$5,266,838), out of which $238,380 (95% UI, $26,401-$465,158) was for indirect cost. The accrued daily total incremental cost was $405,919 (95% UI, $10,409-$877,806).

**Figure 1:**
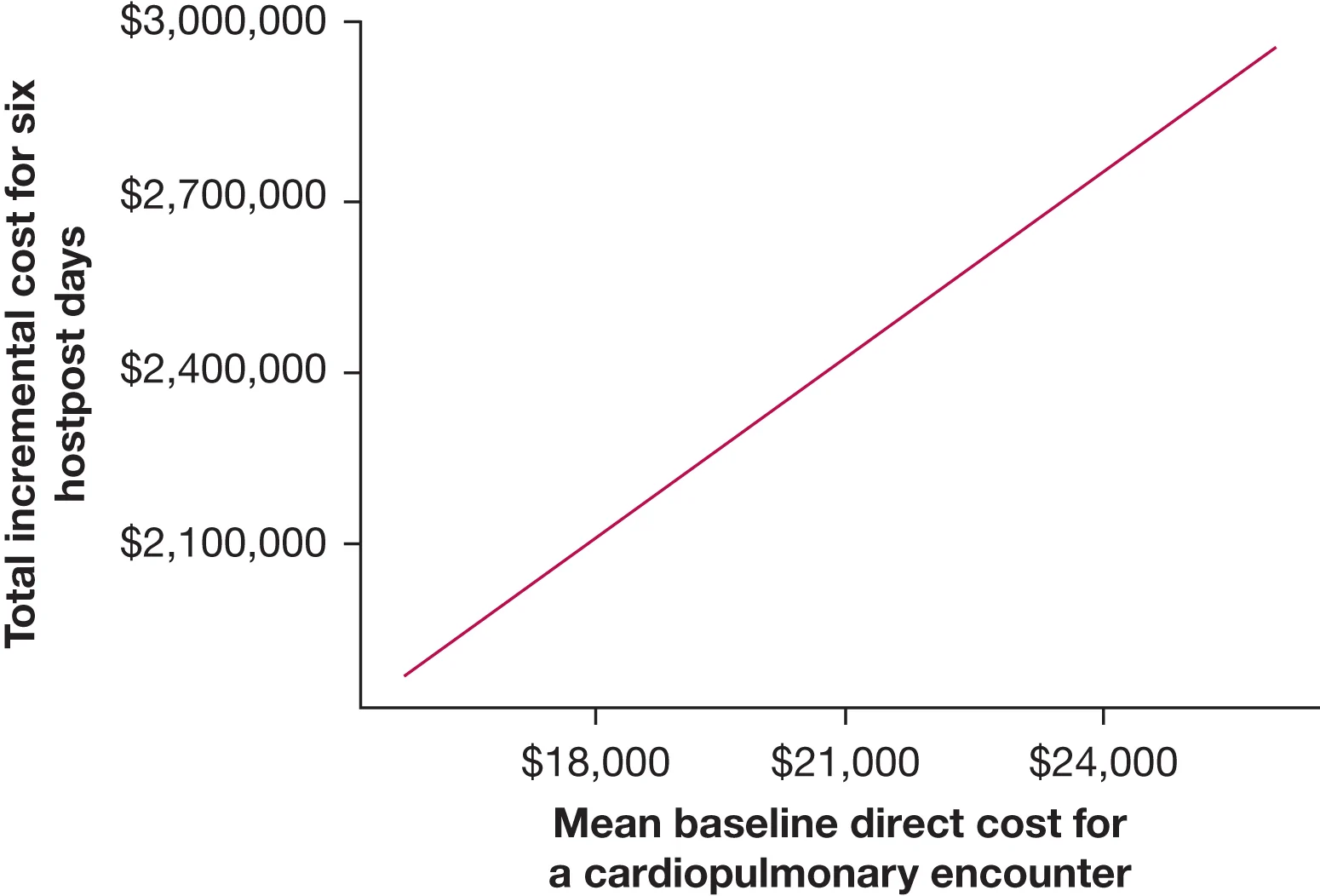
One-way sensitivity analysis for total incremental cost of migrating wildfire smoke for 6 hotspot days, varying mean baseline cardiopulmonary encounter costs, in 12 hospital facilities of the University of Maryland Medical System.

In our 1-way sensitivity analysis, the total cost burden of migrating wildfire smoke in our hospital system was most sensitive to the baseline cost of a cardiopulmonary encounter—a 25% change in this baseline encounter-level cost resulted in a variation of the incremental total burden from $1,857,522 to $2,956,088. Figure 1 shows the change in total incremental cost for different values of mean encounter-level cost, which can help with generalization of the burden estimate in other hospital settings. The incremental cost burden did not significantly vary by changes in mean annual wage, length of stay, and hours of productivity and leisure lost per hospital day.

### Projection of Economic Effects of Future Wildfires

Based on observations from the past 10 years and projected changes in future wildfire frequency and duration, our Poisson distribution informed scenario A (minimum) as 1 wildfire per 10 years, scenario B (expected) as 2 wildfires per 10 years, scenario C (intermediate) as 5 wildfires per 10 years, and scenario D (worst case) as 8 wildfires per 10 years.

For the expected scenario, when future costs were discounted at 3%, the projected total cost of wildfire smoke-related cardiopulmonary encounters was estimated as $6,379,406 (95% UI, $122,412-$13,837,150). For the minimum scenario, the projected total cost was $3,430,192 (95% UI, $59,356-$7,447,571). For the intermediate and worst-case scenarios, the projected total cost was $15,214,218 (95% UI, $309,865-$32,979,738), and $24,046,518 (95% UI, $496,993-$52,117,190), respectively. The future total cost projection for the 4 wildfire scenarios under no discounting and under a 5% discount rate is presented in Table 2.

**Table 2:** Results of the 10-Year Future Cost Projections Under Different Wildfire Scenarios From 12 Hospitals in Maryland^a^

Future Projections	Projected 10-y Total Cost of Wildfire Smoke-Related Cardiopulmonary Encounters
Base case (discounting future costs at 3%)	
Scenario A: 1 wildfire event in 10 y	$3,430,192 (95% UI, $59,356-$7,447,571)
Scenario B: 2 wildfire events in 10 y	$6,379,406 (95% UI, $122,412-$13,837,150)
Scenario C: 5 wildfire events in 10 y	$15,214,218 (95% UI, $309,865-$32,979,738)
Scenario D: 8 wildfire events in 10 y	$24,046,518 (95% UI, $496,993-$52,117,190)
Sensitivity analysis (no discounting)	
Scenario A: 1 wildfire event in 10 y	$4,609,892 (95% UI, $79,770-$10,008,912)
Scenario B: 2 wildfire events in 10 y	$8,028,838 (95% UI, $152,869-$17,416,187)
Scenario C: 5 wildfire events in 10 y	$18,368,861 (95% UI, $370,587-$39,822,052)
Scenario D: 8 wildfire events in 10 y	$28,724,401 (95% UI, $588,004-$62,262,257)
Sensitivity analysis (discounting future costs at 5%)	
Scenario A: 1 wildfire event in 10 y	$2,830,074 (95% UI, $48,972-$6,144,604)
Scenario B: 2 wildfire events in 10 y	$5,508,908 (95% UI, $106,246-$11,948,397)
Scenario C: 5 wildfire events in 10 y	$13,508,899 (95% UI, $276,837-$29,281,190)
Scenario D: 8 wildfire events in 10 y	$21,501,917 (95% UI, $447,191-$46,598,976)

UI = uncertainty interval.

### Modeled Effects of Respirators to Mitigate Cardiopulmonary Complications of Smoke Exposure

From our data, we found that 25% of patients with cardiopulmonary encounters across UMMS facilities during June 2023 were high risk (those with a cardiopulmonary encounter in the previous year). Under the expected scenario, providing respirators with 50%, 75%, and 95% effectiveness to high-risk individuals was projected to yield expected net cost savings of $758,274, $1,162,649, and $1,486,150, respectively. Under the minimum scenario, the corresponding expected net cost savings were $411,148, $628,580, and $802,526. Under the worst-case scenario, they were $2,837,104, $4,361,356, and $5,580,757 (Table 3).

**Table 3:** Projecting Net Cost Savings Under Future Wildfire Scenarios When N95 Respirators Are Provided to High-Risk Individuals

Scenarios	Net Cost Savings (Including Respirator Costs)
Scenario A: 1 wildfire event in 10 y	
Expected effectiveness of N95 respirators (50%)	$411,148 (95% UI, −$16,189 to $920,452) (> 96% of the Monte Carlo simulations yielded a positive return on investment)
High effectiveness of N95 respirators (75%)	$628,580 (95% UI, −$12,427 to $1,392,536) (> 97% of the Monte Carlo simulations yielded a positive return on investment)
Ideal effectiveness of N95 respirators (95%)	$802,526 (95% UI, −$9,417 to $1,770,204) (> 97% of the Monte Carlo simulations yielded a positive return on investment)
Scenario B: 2 wildfire events in 10 y	
Expected effectiveness of N95 respirators (50%)	$758,274 (95% UI, −$34,957 to $1,703,732) (> 96% of the Monte Carlo simulations yielded a positive return on investment)
High effectiveness of N95 respirators (75%)	$1,162,649 (95% UI, −$27,198 to $2,580,836) (> 97% of the Monte Carlo simulations yielded a positive return on investment)
Ideal effectiveness of N95 respirators (95%)	$1,486,150 (95% UI, −$20,990 to $3,282,519) (> 97% of the Monte Carlo simulations yielded a positive return on investment)
Scenario C: 5 wildfire events in 10 y	
Expected effectiveness of N95 respirators (50%)	$1,797,863 (95% UI, −$91,640 to $4,050,092) (> 96% of the Monte Carlo simulations yielded a positive return on investment)
High effectiveness of N95 respirators (75%)	$2,762,256 (95% UI, −$71,998 to $6,140,599) (> 97% of the Monte Carlo simulations yielded a positive return on investment)
Ideal effectiveness of N95 respirators (95%)	$3,533,771 (95% UI, −$56,285 to $7,813,005) (> 97% of the Monte Carlo simulations yielded a positive return on investment)
Scenario D: 8 wildfire events in 10 y	
Expected effectiveness of N95 respirators (50%)	$2,837,104 (95% UI, −$148,393 to $6,395,771) (> 96% of the Monte Carlo simulations yielded a positive return on investment)
High effectiveness of N95 respirators (75%)	$4,361,356 (95% UI, −$116,889 to $9,699,356) (> 97% of the Monte Carlo simulations yielded a positive return on investment)
Ideal effectiveness of N95 respirators (95%)	$5,580,757 (95% UI, −$91,687 to $12,342,225) (> 97% of the Monte Carlo simulations yielded a positive return on investment)

Data represent 12 hospitals in Maryland. UI = uncertainty interval.

For a sensitivity analysis on removing respirators in indoor spaces, we calculated a 0.16 probability that indoor exposure during hotspot days exceeded the EPA threshold of 35 μg/m^3^. This was based on the observed daily average outdoor exposure of 80.78 μg/m^3^ during 6 hotspot days and an infiltration factor of 0.43 needed for indoor PM_2.5_ levels to surpass the EPA threshold. Assuming individuals spend 90% of their day indoors,^31^ we calculated expected net cost savings of $641,814, $987,959, and $1,264,876 for respirators with 50%, 75%, and 95% effectiveness, respectively, under the expected scenario.

## Discussion

In this study, we quantified the economic impact of migrating wildfire smoke in 12 hospital facilities in Maryland, but our results can be generalizable to other states affected by wildfire smoke above the unhealthy air quality levels. We showed that during the 6-day period characterized by polluted air in Baltimore and the surrounding region, elevated cardiopulmonary disease burden across a large medical system increased cost by approximately $2.4 million. Our risk reduction model proposing N95 respirator use by high-risk patients showed the burden of migrating wildfire smoke could be potentially reduced by 13% to 24%.

Our findings support prior modeling studies that have quantified the economic burden of wildfire-related PM_2.5_ on cardiopulmonary health.^32^, ^33^, ^34^, ^35^ Notably, Wei et al^36^ used nationwide Medicare fee-for-service claims from 2000 to 2012 and estimated that a 1-μg/m^3^ short-term increase in PM_2.5_ was associated with approximately $69 million per year in additional inpatient plus postacute care spending for conditions with established PM_2.5_ sensitivity, including cardiovascular and respiratory diagnoses. In contrast, our study provides localized, encounter-level evidence from a real-world integrated health system, illustrating a $2.4 million increase in cardiopulmonary costs attributable to short-term exposure to high levels of migrating wildfire smoke.

Our findings for the economic impact of wildfire smoke are timely. Given accumulating interest on population-based payment systems, our findings can have major implications for such systems. States and hospital systems that operate under the AHEAD model hold health care providers accountable for the health care efficiency, quality, and cost.^11^^,^^37^ These systems have a fixed budget to finance all medical services.^11^^,^^37^ Therefore, high-intensity spending such as that observed in this study uses funds that otherwise could be used to support other clinical operations.

One major implication of our findings for systems operating under AHEAD or fixed global budgets is the need to incorporate risk-adjustment models for unpredictable environmental hazards into global budget calculations. Historically, these budgets have been determined based on historical spending, inflation adjustments, annual growth caps, and quality and performance metrics. Although risk adjustments have been incorporated into global budget calculations, there is a need to account for unexpected environmental hazards, such as foreseeable wildfire smoke.

As a concrete example, a risk-adjustment algorithm could incorporate the following: (1) predicted wildfire smoke exposure for the coming year, (2) system-level vulnerability (eg, population risk profiles, historical cardiopulmonary surge rates during smoke days), and (3) estimated marginal cost per smoke-related encounter. These components could generate a risk-adjusted wildfire smoke contingency budget as part of the global budget. Health systems that adopt cost-reducing interventions—such as targeted respirator deployment, remote monitoring, or proactive outreach as demonstrated in our study—could have these actions integrated into performance metrics. If actual expenditures come in below the predicted risk-adjusted allocation, a share of the savings could be returned to the system as an incentive.

As social/environmental determinants of health are integrated increasingly into electronic health record platforms, our data reinforce the need to consider changes in air quality or natural disasters in these point-of-care initiatives. Establishing actionable notifications to providers focused on providing care to patients at risk of experiencing cardiopulmonary health events is an important step toward personalized medical care and potentially mitigating health care costs.

Our results are subject to limitations inherent in a retrospective study design including the lack of randomization. Because randomization of patients to smoke exposure or to lack of masking is unethical, relying on real-world data is the only feasible approach to address the effects of wildfire smoke. As with any observational study, there is a risk of confounding bias. To address this, we used a semiexperimental study design using a DID approach, a widely used method for quantifying health care resource utilization in observational research. Furthermore, our 10-year projection models relied on several important assumptions. First, we used a simple metric to define high risk among patients with cardiopulmonary disease that was based solely on prior hospitalization. Although prior hospitalization is a strong predictor of future hospitalization, it is possible that robust models accounting for clinical covariates may offer improved specificity of our findings. Second, although we projected the economic effects for a range of numbers of future wildfires in our models, the accuracy of this forecast approach is difficult to assess because of an increase in wildfire events and uncertain future environmental policies in North America. Although our definition of hotspot days was based solely on PM_2.5_ levels exceeding standard thresholds established by the EPA, it is possible that considering alternative PM components, which are known to emanate from wildfires and could affect human health, could affect our results. However, it is notable that PM_2.5_ and ultrafine particles (< 0.1 μm diameter) constitute approximately 90% of the total particle mass of wildfire smoke and are considered to pose the greatest health risks.^38^^,^^39^

## Interpretation

Our findings offer system-level insight into the acute cost burden of extreme PM_2.5_ episodes and highlight the need for preparedness as wildfire smoke events become more frequent. Public health systems that anticipate wildfire smoke migration patterns and proactively initiate preventative measures are needed.

## Funding/Support

Funding from 10.13039/100024377Montgomery County, Maryland, and The University of Maryland Strategic Partnership: MPowering the State, a formal collaboration between the University of Maryland, College Park and the University of Maryland, Baltimore was used to support the investigators’ effort and some of the computational resources needed to complete analyses performed in this study.

## Financial/Nonfinancial Disclosures

The authors have reported to *CHEST* the following: B. A. M. reports personal fees from Actelion Pharmaceuticals, Tenax, Regeneron, and grants from Deerfield Co (outside the scope of this study); and has a patent PCT/US2019/059890 pending to none, a patent #9,605,047 issued to none, a patent PCT/US2020/066886 pending to none, and a patent BWH 2023-152-29618-0438P02 pending to none, all of which are outside the scope of this study. None declared (Z. Z., M. E. M., C. H. B., M. S., C. R., R. M., M. J., A. S., K. Z., W. D.).

## Supplementary Data

e-Online Data
