# Supplementary material for: The Economic Consequence of Increased Health Care Resource Utilization in Maryland Associated With a Major Wildfire Smoke Event
Source: Chest. 2026 Mar 24;170(1):190–9. doi: 10.1016/j.chest.2026.01.034 (PMC13494427; doi:10.1016/j.chest.2026.01.034)
Supplement: Supplementary file 1 [file mmc1.docx]

# Supplemental Information

**The Economic Consequence of Increased Healthcare Resource Utilization in Maryland Associated with a Major Wildfire Smoke Event**

**e-Appendix 1**. Estimating wildfire-related PM_2.5_ levels

As described in our earlier work (1), we used Hazard Mapping System (HMS) data from National Oceanic and Atmospheric Administration (NOAA) (2–4). HMS provides satellite-based measures for smoke density within each polygon (2,5). We, additionally, for each census tract, derived average daily PM_2.5_ concentration from the nearest monitors by the Environmental Protection Agency (EPA)’s Air Quality System (AQS) (6). For each census tract that was not under a smoke plume polygon, we assigned a wildfire-related PM_2.5_ of 0 µg/m³, and the average daily PM_2.5_ concentration was assigned to non-wildfire-related PM_2.5_ for that day. For census tract falling within a smoke plume polygon, the wildfire-related PM_2.5_ was calculated by the difference between the daily average PM_2.5_ concentration and the background level of concentration in the absence of wildfire (corresponding to median concentration during summer months of June to August). For smoke days, non-wildfire-related PM_2.5_ was calculated by the difference of wildfire-related PM_2.5_ and the average concentration on those days (1).

**e-Appendix 2**. Details on the regression analyses, gamma model selection, and parallel-trends testing for the DID analyses

We developed two regression models. Our data contained individual-level encounters for cardiopulmonary conditions at any of our facilities. In the first model, we predicted probability of a cardiopulmonary encounter occurring on hotspot days using a generalized linear model (GLM) with binomial distribution (each individual-level encounter was coded as 1 if it occurred on a hotspot day and 0 otherwise). This allowed us to calculate the probability of a cardiopulmonary encounter occurring on hotspot days of 2023 against calendar-matched orthologous days in the control years (2018 and 2019). We combined all cardiopulmonary-related healthcare utilization encounters, encompassing hospitalizations, ED visits, and outpatient visits to enhance statistical power. In the second model, we conducted a Difference-in-Differences (DID) analysis using a GLM model with a gamma distribution and log link to compare the cost of each cardiopulmonary encounter between hotspot and non-hotspot days in the wildfire year (2023) versus control years (2018–2019). Analogous to a conventional DID design, the treated units are calendar-position days that experienced wildfire smoke exposure in June 2023, with their orthologous days in June 2018–2019 representing pre-treatment observations of the same units. Time is divided into a baseline period (June 2018–2019) and a wildfire year (June 2023), with the ‘post’ indicator defined as an indicator for the wildfire year. The actual wildfire smoke exposure is identified by the interaction between the hotspot indicator and the post indicator.

Given that costs are strictly positive, right skewed, and heteroscedastic, the use of a GLM with a gamma distribution and log link is a standard practice in health economics research (7). We additionally compared the predictive performance of our gamma specification against ordinary least squares (OLS), and the gamma GLM yielded a lower root mean squared error (RMSE) and Akaike information criterion (AIC), indicating a superior predictive performance.

In addition to controlling for available measured confounders in our data, the choice of DID allows for controlling unmeasured temporal factors that changed similarly over the years between the hotspot and non-hotspot days. Examples of such factors include population characteristics, hospital characteristics and hospital staffing, and non-wildfire pollution. We tested the parallel trends assumption of our DID using a placebo test using only 2018 and 2019 data. We found that the placebo interaction (hotspot × year_placebo) was not statistically significant (p = 0.90), suggesting that hotspot-calendar days and non-hotspot days did not differ in costs in pre-intervention years and supporting the parallel-trends assumption.

**e-Appendix 3**. Details on estimating marginal costs of migrating wildfire smoke

We estimated marginal direct medical costs using a non-parametric bootstrap with 1,000 replicates, refitting both models in each replicate and computing marginal costs via g-computation. The direct cost included medical cost of an average cardiopulmonary encounter, including either a hospitalization, ED visit, or outpatient visit. The average direct cost per cardiopulmonary encounter in June 2023 was $20,934 in our UMMS EHR data. Indirect costs were incorporated via a Monte Carlo simulation; in each bootstrap iteration, we drew annual wages from a gamma distribution (mean =$75,585, based on Bureau of Labor Statistics data (8)) with a 95% uncertainty interval (95% UI) extending ±5% of the mean, and combined these draws with bootstrap-sampled lengths of stay (mean 5 days in our data) to compute productivity and leisure losses. In the base case, we assumed 8 hours of productivity loss and 8 hours of leisure loss per hospitalization day, valued at the hourly wage (9–12) and changed the number of hours lost in our sensitivity analyses. We prespecified a directional hypothesis—that wildfire smoke exposure increases total costs—based on prior evidence that PM₂.₅ lead to higher spending (13). Accordingly, we reported both one-sided and two-sided statistical inference, and 95% UI as suggested by Ruxton and Neuhäuser (14).

All costs were adjusted to and reported in 2024 U.S. dollars (USD). To enhance the generalizability of our findings to other healthcare systems and settings, we also performed a one-way sensitivity analysis. We estimated the economic burden of migrating wildfire smoke by varying the mean baseline direct medical cost of a healthcare encounter due to cardiopulmonary conditions, the mean annual wage of an American, and the length of stay per encounter by ±25%. The 25% variation in average annual wage reflects the range of wages observed across U.S. states. We also varied the number of hours lost per cardiopulmonary encounter from 8 to 20 hours per day.

**e-Appendix 4**. Details on projecting the economic effects of future wildfires

For projections of future severe wildfire events, we first calculated the baseline frequency using 10 years of historical data (2014–2023) from the EPA AQS (6) and NOAA’s HMS (2,5). We identified one major event during this baseline period (the event analyzed in this study) that produced two stretches of three consecutive hotspot days each.

To project the expected frequency of severe wildfires over the next decade, we used a Bayesian approach with a Jeffreys prior for the Gamma distribution to estimate the posterior distribution of the mean annual rate of severe events. To incorporate evidence of historical growth in wildfire activity, we applied decadal trends derived from Westerling (15), from which we calculated an average increase of approximately 35% in frequency and 50% in event duration per decade (based on changes from 1983–1992 to 2003–2012).

We implemented a two-loop Monte Carlo simulation framework. In the first loop, we drew samples from the posterior distribution and multiplied each by 1.35 to represent the projected 35% increase in event frequency. Using the resulting mean rate, we then simulated the number of future wildfire events using a Poisson distribution. We defined four projection scenarios: Scenario A (Minimum)—same number of wildfire events as in the past 10 years; Scenario B (Expected)–mean of the Poisson distribution; Scenario C (Intermediate)–midpoint between Scenarios B and D; and Scenario D (Worst-case)–97.5th percentile of the Poisson distribution. For each simulated future event, duration was scaled relative to the baseline period by applying the 50% per-decade increase, adjusted for the calendar year of occurrence (15).

We further adjusted for population growth in Maryland using projections from the Maryland Department of Planning’s State Data and Analysis Center (16). A smoothing spline was fitted to the projected population trend for the next decade, and the expected number of cardiopulmonary encounters was scaled in direct proportion to population changes. Finally, we accounted for real medical cost growth using a 1.91% annual real growth rate derived from published literature (17). Together, these components provided an integrated projection of future cardiopulmonary health and economic impacts associated with severe wildfire events under the minimum, expected, intermediate, and worst-case scenarios.

For the wildfire events occurring in the future, a 3% discount rate was applied to projected costs in line with recommendations of the Second US Panel on Cost-Effectiveness in Health and Medicine (9). In a sensitivity analysis, we also reported the results without any discounting and with a discounting rate of 5% for future costs.

**e-Appendix 5**. Calculating the net cost savings associated with providing N95 respirators to high-risk individuals

We modeled the potential net cost savings of providing N95 respirators to high-risk cardiopulmonary patients during hotspot days. High-risk individuals were defined as those with a cardiopulmonary encounter in our medical system in the prior year. These high-risk cases can be detected using a centralized clinical database. An automated hospital system could then send targeted messages to these individuals before anticipated wildfire smoke exposure, providing instructions on proper respirator use. We reasoned that a significant proportion of cardiopulmonary encounters due to wildfire smoke are from recurrent cases (18–21) and could be prevented by (i) identifying high-risk patients based on clinical history and (ii) providing these patients instructions to use protective tools. Based on the literature, we assumed an expected effectiveness of N95 respirators that was ‘expected’ (50%), ‘high’ (75%), or ‘ideal’ (95%) (22). In this framework, the percentage indicates the expected time‐average PM_2.5_ exposure reduction after accounting for real-world imperfections in respirator usage, including incomplete adherence, potential respirator damage, improper facial fit, and inconsistent use over a full 24-hr period (22).

Among patients with a cardiopulmonary encounter during the smoke-exposure period (June 2023), we calculated the proportion who also had a cardiopulmonary encounter in the previous year. We then assumed that this proportion of the attributable cost of smoke exposure could be prevented—proportional to respirator effectiveness—if hospitals provided respirators to all high-risk individuals identified from the previous year’s records. We assumed a unit cost of $0.78 per respirator when purchased at bulk based on data from Federal Emergency Management Agency (3M 9105 respirator) (23), converted to 2024 USD using the Consumer Price Index (24). We assumed a replacement cost of respirators for subsequent events.

We additionally conducted a sensitivity analysis. Recognizing that patients may not always be aware of unsafe indoor pollution levels and may unknowingly remove their respirators in such situations, we incorporated estimates of infiltration from literature. Based on Liang et al. (25), the mean infiltration factor during fire days was 0.23 (25th percentile: 0.16; 75th percentile: 0.36). Using these values, we parameterized a log-normal distribution for infiltration. Using this distribution, we calculated the probability of exposure time with infiltration factor needed to make the indoor PM₂.₅ levels surpassing the EPA threshold of 35 µg/m³. We then adjusted the protection effect of respirators based on average time spent inside and the probability of indoor exposure time during hotspot days exceeding the EPA’s unhealthy threshold.

**e-Table 1**. ICD-10 codes for identifying patients with a cardiopulmonary encounter

| **Cardiac Disease** | **Pulmonary Disease** | **Heart Failure Disease** |
| --- | --- | --- |
| I00-I49.9 | J00-J95.09 | E87.70 |
| I51.0-I52 | J96-J99 | E87.79 |
| I5A |  | I50-I50.9 |
|  |  | I11.0 |
|  |  | I13.0 |
|  |  | I13.2 |
|  |  | I27.81 |

**e-Table 2**. Baseline characteristics of cardiopulmonary events during the hotspot days of 2023 relative to their orthologous days in control years (2018-2019).

| **Variable** | **Sample size (N)** | **2018-2019**  **(**N = 806) | **2023**  **(**N = 588) | **p-value** |
| --- | --- | --- | --- | --- |
| Age (mean [SD]) | 1,394 | 66 (15) | 67 (14) | 0.06 |
| *Race* (N [%]) | 1,394 |  |  | 0.07 |
| White |  | 515 (64%) | 390 (66%) |  |
| Black or African American |  | 234 (29%) | 174 (30%) |  |
| Asian |  | 23 (2.9%) | 6 (1.0%) |  |
| Other/Unknown |  | 34 (4.2%) | 18 (3.1%) |  |
| *Sex* (N [%]) | 1,394 |  |  | 0.28 |
| Female |  | 372 (46%) | 289 (49%) |  |
| Male |  | 434 (54%) | 299 (51%) |  |
| BMI (mean [SD]) | 1,353 | 30 (14) | 30 (8) | 0.71 |
| *Smoking status* (N [%]) | 1,282 |  |  | 0.00 |
| Never |  | 238 (31%) | 169 (32%) |  |
| Former |  | 242 (32%) | 147 (28%) |  |
| Smoker, light smoker |  | 21 (2.8%) | 9 (1.7%) |  |
| Smoker, heavy smoker |  | 109 (15%) | 58 (11%) |  |
| Smoker, current status unknown |  | 140 (19%) | 149 (28%) |  |
| *Weekday* (N [%]) | 1,394 |  |  | 0.84 |
| Tuesday |  | 150 (19%) | 104 (18%) |  |
| Wednesday |  | 279 (35%) | 215 (37%) |  |
| Thursday |  | 240 (30%) | 177 (30%) |  |
| Friday |  | 137 (17%) | 92 (16%) |  |

SD: standard deviation; BMI: body mass index.

**e-Table 3**. Results of the two regression models from the multiple imputation sensitivity analysis.

| **Variable** | **Estimate** | **SE** | **P Value** |
| --- | --- | --- | --- |
| **Model 1 (predicting rate of cardiopulmonary events occurring on hotspot days)** | | | |
| Intercept | -1.409 | 0.246 | 0.000 |
| Year (2023 vs. 2018-2019) | 0.192 | 0.086 | 0.026 |
| *Demographics* |  |  |  |
| Age | 0.003 | 0.002 | 0.257 |
| Sex (Male vs. Female) | 0.080 | 0.074 | 0.277 |
| *Race/ethnicity (reference White)* |  |  |  |
| Black or African American | -0.004 | 0.087 | 0.962 |
| Asian | 0.774 | 0.301 | 0.010 |
| Other | 0.505 | 0.202 | 0.012 |
| *Smoking (reference never smoker)* |  |  |  |
| Former | -0.069 | 0.097 | 0.481 |
| Smoker, light smoker | -0.171 | 0.254 | 0.502 |
| Smoker, heavy smoker | -0.068 | 0.120 | 0.570 |
| Smoker, current status unknown | 0.021 | 0.128 | 0.868 |
| BMI | 0.002 | 0.005 | 0.708 |
| **Model 2 (predicting unit cost of cardiopulmonary events falling on hotspot days)** | | | |
| Intercept | 9.464 | 0.161 | 0.000 |
| Post (Wildfire year-June 2023 indicator) | 0.088 | 0.062 | 0.154 |
| Hotspot (Treated unit indicator) | -0.029 | 0.079 | 0.712 |
| *Demographics* |  |  |  |
| Age | 0.001 | 0.001 | 0.495 |
| Sex (Male vs. Female) | 0.125 | 0.048 | 0.009 |
| *Race/ethnicity (reference White)* |  |  |  |
| Black or African American | -0.135 | 0.056 | 0.016 |
| Asian | 0.199 | 0.205 | 0.333 |
| Other | -0.086 | 0.133 | 0.519 |
| *Smoking (reference never smoker)* |  |  |  |
| Former | 0.038 | 0.061 | 0.534 |
| Smoker, light smoker | -0.389 | 0.151 | 0.010 |
| Smoker, heavy smoker | -0.156 | 0.075 | 0.037 |
| Smoker, current status unknown | -2.605 | 0.090 | 0.000 |
| BMI | 0.001 | 0.003 | 0.681 |
| Hotspot*Post (DID estimator) | 0.063 | 0.113 | 0.578 |

All the models adjusted for weekday and 12 hospital facilities, including Baltimore Washington Medical Center, Charles Regional Medical Center, Harford Memorial Hospital, University of Maryland Capital Region Health, University of Maryland Shore Medical Center at Chestertown, University of Maryland Shore Medical Center at Dorchester, University of Maryland Shore Medical Center at Easton, University of Maryland Medical Center, University of Maryland Medical Center Midtown Campus, University of Maryland Rehabilitation and Orthopedic Institute, University of Maryland St. Joseph Medical Center, and Upper Chesapeake Medical Center.

SE: standard error; BMI: body mass index; DID: difference-in-difference

**e-Table 4**. Results of the two regression models for cardiopulmonary hospitalizations.

| **Variable** | **Estimate** | **SE** | **P Value** |
| --- | --- | --- | --- |
| **Model 1 (predicting rate of cardiopulmonary events occurring on hotspot days)** | | | |
| Intercept | -1.409 | 0.311 | 0.000 |
| Year (2023 vs. 2018-2019) | 0.147 | 0.111 | 0.186 |
| *Demographics* |  |  |  |
| Age | 0.004 | 0.003 | 0.198 |
| Sex (Male vs. Female) | 0.076 | 0.097 | 0.431 |
| *Race/ethnicity (reference White)* |  |  |  |
| Black or African American | -0.030 | 0.113 | 0.792 |
| Asian | 0.780 | 0.371 | 0.036 |
| Other | 0.598 | 0.265 | 0.024 |
| *Smoking (reference never smoker)* |  |  |  |
| Former | -0.067 | 0.109 | 0.541 |
| Smoker, light smoker | -0.086 | 0.340 | 0.799 |
| Smoker, heavy smoker | -0.098 | 0.142 | 0.493 |
| Smoker, current status unknown | 0.211 | 0.519 | 0.683 |
| BMI | 0.003 | 0.006 | 0.558 |
| **Model 2 (predicting unit cost of cardiopulmonary events falling on hotspot days)** | | | |
| Intercept | 9.636 | 0.160 | 0.000 |
| Post (Wildfire year-June 2023 indicator) | 0.061 | 0.062 | 0.329 |
| Hotspot (Treated unit indicator) | -0.042 | 0.079 | 0.592 |
| *Demographics* |  |  |  |
| Age | 0.000 | 0.001 | 0.945 |
| Sex (Male vs. Female) | 0.116 | 0.048 | 0.017 |
| *Race/ethnicity (reference White)* |  |  |  |
| Black or African American | -0.144 | 0.057 | 0.011 |
| Asian | 0.130 | 0.194 | 0.503 |
| Other | -0.219 | 0.137 | 0.111 |
| *Smoking (reference never smoker)* |  |  |  |
| Former | 0.002 | 0.055 | 0.965 |
| Smoker, light smoker | -0.196 | 0.149 | 0.189 |
| Smoker, heavy smoker | -0.143 | 0.070 | 0.042 |
| Smoker, current status unknown | -0.482 | 0.268 | 0.072 |
| BMI | 0.004 | 0.003 | 0.188 |
| Hotspot*Post (DID estimator) | 0.094 | 0.118 | 0.424 |

All the models adjusted for weekday and 12 hospital facilities, including Baltimore Washington Medical Center, Charles Regional Medical Center, Harford Memorial Hospital, University of Maryland Capital Region Health, University of Maryland Shore Medical Center at Chestertown, University of Maryland Shore Medical Center at Dorchester, University of Maryland Shore Medical Center at Easton, University of Maryland Medical Center, University of Maryland Medical Center Midtown Campus, University of Maryland Rehabilitation and Orthopedic Institute, University of Maryland St. Joseph Medical Center, and Upper Chesapeake Medical Center.

SE: standard error; BMI: body mass index; DID: difference-in-difference.

**e-Table 5**. Results of the two regression models for cardiopulmonary emergency department visits.

| **Variable** | **Estimate** | **SE** | **P Value** |
| --- | --- | --- | --- |
| **Model 1 (predicting rate of cardiopulmonary events occurring on hotspot days)** | | | |
| Intercept | -1.447 | 0.312 | 0.000 |
| Year (2023 vs. 2018-2019) | 0.153 | 0.114 | 0.180 |
| *Demographics* |  |  |  |
| Age | 0.004 | 0.003 | 0.161 |
| Sex (Male vs. Female) | 0.121 | 0.097 | 0.213 |
| *Race/ethnicity (reference White)* |  |  |  |
| Black or African American | 0.009 | 0.115 | 0.939 |
| Asian | 0.020 | 0.413 | 0.961 |
| Other | 0.574 | 0.257 | 0.026 |
| *Smoking (reference never smoker)* |  |  |  |
| Former | -0.106 | 0.112 | 0.344 |
| Smoker, light smoker | -0.381 | 0.320 | 0.234 |
| Smoker, heavy smoker | -0.046 | 0.136 | 0.735 |
| Smoker, current status unknown | 0.327 | 0.530 | 0.537 |
| BMI | 0.000 | 0.006 | 0.970 |
| **Model 2 (predicting unit cost of cardiopulmonary events falling on hotspot days)** | | | |
| Intercept | 9.045 | 0.139 | 0.000 |
| Post (Wildfire year-June 2023 indicator) | 0.079 | 0.055 | 0.150 |
| Hotspot (Treated unit indicator) | -0.048 | 0.072 | 0.503 |
| *Demographics* |  |  |  |
| Age | 0.004 | 0.001 | 0.002 |
| Sex (Male vs. Female) | 0.123 | 0.042 | 0.004 |
| *Race/ethnicity (reference White)* |  |  |  |
| Black or African American | -0.075 | 0.051 | 0.136 |
| Asian | 0.423 | 0.192 | 0.028 |
| Other | 0.057 | 0.122 | 0.642 |
| *Smoking (reference never smoker)* |  |  |  |
| Former | 0.047 | 0.049 | 0.334 |
| Smoker, light smoker | -0.071 | 0.123 | 0.565 |
| Smoker, heavy smoker | 0.013 | 0.059 | 0.826 |
| Smoker, current status unknown | -0.264 | 0.246 | 0.284 |
| BMI | 0.007 | 0.003 | 0.005 |
| Hotspot*Post (DID estimator) | 0.146 | 0.103 | 0.157 |

All the models adjusted for weekday and 12 hospital facilities, including Baltimore Washington Medical Center, Charles Regional Medical Center, Harford Memorial Hospital, University of Maryland Capital Region Health, University of Maryland Shore Medical Center at Chestertown, University of Maryland Shore Medical Center at Dorchester, University of Maryland Shore Medical Center at Easton, University of Maryland Medical Center, University of Maryland Medical Center Midtown Campus, University of Maryland Rehabilitation and Orthopedic Institute, University of Maryland St. Joseph Medical Center, and Upper Chesapeake Medical Center.

SE: standard error; BMI: body mass index; DID: difference-in-difference.

**e-Table 6**. Results of the two regression models for cardiopulmonary outpatient visits.

| **Variable** | **Estimate** | **SE** | **P Value** |
| --- | --- | --- | --- |
| **Model 1 (predicting rate of cardiopulmonary events occurring on hotspot days)** | | | |
| Intercept | 0.240 | 0.935 | 0.797 |
| Year (2023 vs. 2018-2019) | 0.475 | 0.222 | 0.033 |
| *Demographics* |  |  |  |
| Age | -0.009 | 0.008 | 0.256 |
| Sex (Male vs. Female) | 0.116 | 0.173 | 0.502 |
| *Race/ethnicity (reference White)* |  |  |  |
| Black or African American | -0.232 | 0.230 | 0.314 |
| Asian | 0.313 | 0.915 | 0.733 |
| Other | -0.532 | 0.651 | 0.413 |
| *Smoking (reference never smoker)* |  |  |  |
| Former | -0.691 | 0.648 | 0.286 |
| Smoker, light smoker | -0.182 | 0.695 | 0.793 |
| Smoker, heavy smoker | -0.071 | 0.720 | 0.921 |
| Smoker, current status unknown | -0.331 | 0.523 | 0.527 |
| BMI | -0.016 | 0.013 | 0.212 |
| **Model 2 (predicting unit cost of cardiopulmonary events falling on hotspot days)*** | | | |
| Intercept | 1.190 | 0.358 | 0.001 |
| Post (Wildfire year-June 2023 indicator) | 0.118 | 0.095 | 0.212 |
| Hotspot (Treated unit indicator) | 0.130 | 0.111 | 0.242 |
| *Demographics* |  |  |  |
| Age | 0.006 | 0.003 | 0.057 |
| Sex (Male vs. Female) | -0.027 | 0.067 | 0.693 |
| *Race/ethnicity (reference White)* |  |  |  |
| Black or African American | 0.007 | 0.086 | 0.939 |
| Asian | -0.326 | 0.305 | 0.286 |
| Other | -0.204 | 0.239 | 0.393 |
| *Smoking (reference never smoker)* |  |  |  |
| Former | -0.846 | 0.253 | 0.001 |
| Smoker, light smoker | -1.122 | 0.290 | 0.000 |
| Smoker, heavy smoker | -0.703 | 0.288 | 0.015 |
| Smoker, current status unknown | -1.418 | 0.213 | 0.000 |
| BMI | 0.008 | 0.005 | 0.109 |
| Hotspot*Post (DID estimator) | 0.266 | 0.147 | 0.071 |

*This regression was performed on a linear transformation of costs (f(cost)=cost/1000+1) to facilitate model convergence.

All the models adjusted for weekday and 12 hospital facilities, including Baltimore Washington Medical Center, Charles Regional Medical Center, Harford Memorial Hospital, University of Maryland Capital Region Health, University of Maryland Shore Medical Center at Chestertown, University of Maryland Shore Medical Center at Dorchester, University of Maryland Shore Medical Center at Easton, University of Maryland Medical Center, University of Maryland Medical Center Midtown Campus, University of Maryland Rehabilitation and Orthopedic Institute, University of Maryland St. Joseph Medical Center, and Upper Chesapeake Medical Center.

SE: standard error; BMI: body mass index; DID: difference-in-difference.

**References**

1.	Maldarelli ME, Song H, Brown CH, Situt M, Reilly C, Mahurkar AA, et al. Polluted Air from Canadian Wildfires and Cardiopulmonary Disease in the Eastern US. JAMA Netw Open. 2024 Dec 2;7(12):e2450759.

2.	National Oceanic and Atmospheric Administration-Office of Satellite and Product Operations. NOAA OSPO. [cited 2025 Sep 30]. Hazard Mapping System Fire and Smoke Product. Available from: https://www.ospo.noaa.gov/products/land/hms.html

3.	Ruminski M, Kondragunta S, Draxler R, Zeng J. Recent Changes to the Hazard Mapping System. Proc 15th Int Emiss Inventory Conf 2006 May 15 Vol 15 P 18 Natl Ocean Atmospheric Adm.

4.	Schroeder W, Ruminski M, Csiszar I, Giglio L, Prins E, Schmidt C, et al. Validation analyses of an operational fire monitoring product: The Hazard Mapping System. Int J Remote Sens. 2008 Oct 20;29(20):6059–66.

5.	United States Census Bureau. TIGER/Line Shapefile, Current, Nation, U.S., State and Equivalent Entities [Internet]. U.S. Department of Commerce, U.S. Census Bureau, Geography Division (Point of Contact); [cited 2025 Sep 30]. Available from: https://catalog.data.gov/dataset/tiger-line-shapefile-current-nation-u-s-state-and-equivalent-entities

6.	United States Environmental Protection Agency. Air Quality System (AQS) API [Internet]. [cited 2025 Sep 30]. Available from: https://aqs.epa.gov/aqsweb/documents/data_api.html

7.	Barber J, Thompson S. Multiple regression of cost data: use of generalised linear models. J Health Serv Res Policy. 2004 Oct;9(4):197–204.

8.	Bureau of Labor Statistics. Bureau of Labor Statistics. [cited 2025 Nov 15]. All States and U.S. 2024 Annual Averages. Available from: https://data.bls.gov/cew/apps/table_maker/v4/table_maker.htm#type=0&year=2024&qtr=A&own=0&ind=10&supp=1

9.	Sanders GD, Neumann PJ, Basu A, Brock DW, Feeny D, Krahn M, et al. Recommendations for Conduct, Methodological Practices, and Reporting of Cost-effectiveness Analyses: Second Panel on Cost-Effectiveness in Health and Medicine. JAMA. 2016 Sep 13;316(10):1093–103.

10.	Navrud S. Valuing Health Impacts from Air Pollution in Europe. Environ Resour Econ. 2001 Dec 1;20(4):305–29.

11.	Rozan A. How to measure health costs induced by air pollution? Swiss J Econ Stat SJES. 2001;137(I):103–16.

12.	Zafari Z, Park JE, Muennig PA. Different considerations in economic impacts of aircraft noise. J Expo Sci Environ Epidemiol. 2024 Jun 4;

13.	Deryugina T, Heutel G, Miller NH, Molitor D, Reif J. The Mortality and Medical Costs of Air Pollution: Evidence from Changes in Wind Direction. Am Econ Rev. 2019 Dec;109(12):4178–219.

14.	Ruxton GD, Neuhäuser M. When should we use one-tailed hypothesis testing? Methods Ecol Evol. 2010;1(2):114–7.

15.	Westerling AL. Increasing western US forest wildfire activity: sensitivity to changes in the timing of spring. Philos Trans R Soc B Biol Sci. 2016 Jun 5;371(1696):20150178.

16.	Maryland Department of Planning. Planning. [cited 2025 Sep 30]. Maryland State Data Center. Available from: https://planning.maryland.gov/MSDC/Pages/default.aspx

17.	Kazi DS, Elkind MSV, Deutsch A, Dowd WN, Heidenreich P, Khavjou O, et al. Forecasting the Economic Burden of Cardiovascular Disease and Stroke in the United States Through 2050: A Presidential Advisory From the American Heart Association. Circulation [Internet]. 2024 Jul 23 [cited 2025 Sep 30]; Available from: https://www.ahajournals.org/doi/10.1161/CIR.0000000000001258

18.	Behrouzi B, Claggett B, Havighurst T, Mao L, Joseph J, Austin P, et al. Abstract 19059: Risk Factors for the First Cardiopulmonary Hospitalization Differ From Those of Recurrent Cardiopulmonary Hospitalizations in Patients With Cardiovascular Disease Vaccinated Against Influenza —A Secondary Analysis of the INVESTED Trial. Circulation. 2023 Nov 7;148(Suppl_1):A19059–A19059.

19.	Nichols GA, Bell TJ, Pedula KL, O’Keeffe-Rosetti M. Medical care costs among patients with established cardiovascular disease. Am J Manag Care. 2010 Mar;16(3):e86–93.

20.	Nghiem S, Afoakwah C, Scuffham P, Byrnes J. A baseline profile of the Queensland Cardiac Record Linkage Cohort (QCard) study. BMC Cardiovasc Disord. 2022 Feb 5;22(1):35.

21.	Lindenauer PK, Dharmarajan K, Qin L, Lin Z, Gershon AS, Krumholz HM. Risk Trajectories of Readmission and Death in the First Year after Hospitalization for Chronic Obstructive Pulmonary Disease. Am J Respir Crit Care Med. 2018 Apr 15;197(8):1009–17.

22.	Brook RD, Rajagopalan S, Al‐Kindi S. N95 Respirators for Cardiovascular Protection During Wildfire Smoke Events. J Am Heart Assoc. 2024 Jan 16;13(2):e032422.

23.	Federal Emergency Management Agency. Respirators for Healthcare during COVID-19: Authorized Use and Avoiding Fraudulent Products [Internet]. 2021 [cited 2025 Nov 24]. Available from: https://www.fema.gov/fact-sheet/respirators-healthcare-during-covid-19-authorized-use-and-avoiding-fraudulent-products

24.	Bureau of Labor Statistics [Internet]. [cited 2025 Nov 24]. Consumer Price Index Inflation Calculator. Available from: https://www.bls.gov/data/inflation_calculator.htm

25.	Liang Y, Sengupta D, Campmier MJ, Lunderberg DM, Apte JS, Goldstein AH. Wildfire smoke impacts on indoor air quality assessed using crowdsourced data in California. Proc Natl Acad Sci. 2021 Sep 7;118(36):e2106478118.
